# Combination Therapy with Bisoprolol and Tissue Protective Molecule ARA 284 Is Cardio-Protective and Improves Survival in Experimental Cancer Cachexia

**DOI:** 10.3390/jcdd13060241

**Published:** 2026-06-01

**Authors:** Masatsugu Okamura, Sandra Palus, Wolfram Döehner, Stephan von Haehling, Stefan D. Anker, Michael Brines, Jochen Springer

**Affiliations:** 1 Berlin Institute of Health Center for Regenerative Therapies (BCRT), Charité—Universitätsmedizin Berlin, 13353 Berlin, Germany; 2 Department of Rehabilitation Medicine, School of Medicine, Yokohama City University, Yokohama 236-0004, Japan; 3 Deutsches Herzzentrum der Charité, Department of Cardiology, Angiology and Intensive Care Medicine, Augustenburger Platz 1, 13353 Berlin, Germany; 4 Center for Stroke Research Berlin, Charité—Universitätsmedizin Berlin, 13353 Berlin, Germany; 5 German Centre for Cardiovascular Research (DZHK) Partner Site Berlin, 13092 Berlin, Germany; 6 Department of Cardiology (CVK), Charité—Universitätsmedizin Berlin, 13353 Berlin, Germany; 7 Department of Cardiology and Pneumology, University of Göttingen Medical Center (UMG), 37075 Goettingen, Germany; 8 Feinstein Institutes for Medical Research, Northwell Health, Manhasset, NY 11030, USA

**Keywords:** cancer cachexia, tissue-protective molecule, muscle wasting, survival, cardiac function, hepatoma

## Abstract

Background: Cancer cachexia is a serious condition during the last stages of the disease, which is characterized by the loss of muscle and fat mass in patients with cancer. There are no effective treatments for cancer cachexia, and new treatment interventions are urgently needed. We have previously demonstrated that 5 mg/kg/day bisoprolol and 1.7 µg/kg/day ARA 284, a small non-erythropoietic tissue protective peptide, separately have positive effects in a rat model of cancer cachexia. Methods: We investigated the compound effects of both bisoprolol and ARA 284 by targeting multiple pathways in the Yoshida hepatoma rat model of cancer cachexia. Rats were randomly allocated to one of the following treatment groups: bisoprolol (5 mg/kg/day), ARA 284 (1.7 µg/kg/day), a 25% combination (1.25 mg/kg/day bisoprolol + 0.425 µg/kg/day ARA 284), a 75% combination (3.75 mg/kg/day bisoprolol + 1.275 µg/kg/day ARA 284), or placebo. Results: The combination of 3.75 mg/kg/day bisoprolol and 1.275 µg/kg/day ARA 284 showed the strongest overall effects compared with the respective effective monotherapies, respectively, or placebo across multiple endpoints, including body weight, lean mass, food intake, spontaneous activity, and cardiac function in a rat model of cancer cachexia (*p* < 0.01, respectively). Furthermore, this combination therapy had the strongest effects on survival against the placebo (hazard ratio 0.08, 95% confidence interval 0.04 to 0.17, *p* < 0.001). Conclusions: Our findings show that the combination of bisoprolol and ARA 284 is beneficial in a hepatoma cachexia model and may provide greater overall effects than either monotherapy alone.

## 1. Introduction

Cancer cachexia is a severe complication during the last stages of the disease, characterized by the substantial loss of muscle and fat mass in cancer patients [1,2]. Cachexia was described as “a complex metabolic syndrome caused by underlying illnesses and characterized by loss of muscle mass with or without loss of fat mass” at a consensus conference in Washington, USA, in 2006 [3]. The widely accepted diagnostic criteria are a weight loss of at least 5% over 12 months or less in the presence of underlying illness, plus three or more of the following five criteria: decreased muscle strength, fatigue, anorexia, low fat-free mass index, and abnormal biochemistry [3]. Cancer cachexia is a common condition, affecting approximately half of patients with advanced cancer [4,5]. Cancer cachexia is also associated with functional impairment, poor quality of life, increased side effects and treatment interruptions, and reduced survival [5,6]. Recent evidence indicates that cancer cachexia affects not only skeletal muscle but also cardiac muscle, leading to impaired cardiac function and reduced survival [7]. Unfortunately, at present, there are no effective treatments for cancer cachexia, and new treatment interventions are urgently needed. Recent guideline updates continue to emphasize that cancer cachexia remains a major unmet clinical need and that effective pharmacological treatment options are limited [8,9].

Several preclinical studies in recent years have suggested that β-blockers may have potential therapeutic effects on certain aspects of cancer cachexia [10], although no clinically approved treatments currently exist. Bisoprolol is a highly selective β1-adrenoceptor antagonist. We have previously shown that 5 mg/kg/day bisoprolol significantly improved body weight, body composition, cardiac function, and even survival in a rat model of cancer cachexia [11]. In addition, we have also shown that bisoprolol partially suppressed severe catabolic profiles across multiple pathways. In a previous study conducted in patients with heart failure, carvedilol, a non-selective beta blocker, has been found to have protective effects on human muscle [12]. The randomized controlled COPERNICS trial administered carvedilol to patients with heart failure and reported that the intervention group maintained body weight and had less cachexia than the placebo group [12].

The erythropoietic protein hormone erythropoietin (EPO) is released mainly from the peritubular interstitial cells in the kidney [13]. In clinical practice, EPO is prescribed as a treatment for the anemia of renal failure or cancer, which contributes to fatigue, poor quality of life, and reduced survival. In addition to the kidney, EPO is produced by many other cells of the body, including the brain and liver, and functions to protect tissues from inflammation and cellular injury, as well as restore damaged tissue. We have previously shown that EPO treatment in tumor-bearing rats improved body weight, body composition, and survival, and attenuated cardiac wasting, as reflected by preservation of cardiac muscle mass and cardiac function [14]. However, EPO is known to be effective in these non-erythropoietic roles only at high concentrations, and therefore treatment can lead to serious side effects such as polycythemia, hypertension, and thrombosis. It has been found that peptide sequences based on the structure of EPO have tissue protective properties of the EPO molecule but does not support erythropoiesis. We have reported that a partially overlapping peptide derived from helix A of EPO, ARA 284 (amino acids 14–28), maintained cardiac mass and function, which contributed to improving survival in a rat model of cancer cachexia [15]. Although these treatments have been examined separately, a combination therapy is likely to have positive effects in the multi-factorial cancer cachexia by targeting multiple pathways. In the present study, we investigated whether the combination of bisoprolol and ARA 284 could provide greater therapeutic benefit on body composition, cardiac function, and mortality in the Yoshida hepatoma rat model of cancer cachexia by targeting multiple pathways.

## 2. Materials and Methods

### 2.1. Study Design

Eight-week-old male Wistar Han rats (*n* = 175) were housed in groups of three or four at a constant temperature of 22 °C with a 12 h light cycle under SPF conditions and their cages randomly located on racks. Their mean weight was 206.8 ± 11.3 g. Food and water were freely available to animals. After an accumulation period of 7 days, rats were randomized to control (no tumor) or the intraperitoneally implanted 10^8^ Yoshida hepato-ma AH-130 cells. On Day 16 or the day of death if the rats had to be euthanized earlier to meet ethical requirements, blood samples and organs were taken. The weights of all organs were recorded. Additionally, before tumor injection and on the day of sacrifice, nuclear magnetic resonance was used to determine body weight and body composition. On Days 0 and 10/11, cardiac function (echocardiography) and quality of life indicators (food intake and spontaneous activity) were measured. At the end of the study on Day 16 or the day tumor-bearing rats had to be euthanized because they had met ethical endpoints, tumor cells were counted. All procedures were approved by the local animal use and care committee (G 0114/08 LaGeSo, Berlin, Germany). A priori ethical endpoints in accordance with UKCCCR recommendations: (1) persistent anorexia / dehydration; (2) loss of >20% of body weight; (3) apathy, no reaction to light stimulation; (4) hypothermia; (5) labored breathing, cyanosis; (6) persistent diarrhea. All study personnel were blinded to treatment allocation and all analyses were performed by investigators blinded to treatment allocations. The ascites volume was recorded and tumor cell number was determined using a Neubauer chamber using a standard protocol [11]. The compounds were administered by daily gavage (sham, placebo, and bisoprolol) or by intraperitoneal injection (ARA 284).

### 2.2. ARA 284 Peptide

ARA 284 is a 15-amino-acid peptide with a molecular weight of 1708, derived from positions 14 to 28 of helix A of EPO (RYLLEAKEAENITTG). The peptide was synthesized using standard F-moc solid-phase peptide synthesis and purified through HPLC and ion-exchange chromatography. It was stored as a lyophilized powder. Its tissue-protective properties were verified using a standardized in vitro screening assay with human umbilical vein endothelial cells exposed to the toxin staurosporine [15]. Further in vivo assessments, such as protection from renal ischemia-reperfusion and sciatic nerve crush injuries, confirmed that ARA 284 is a tissue-protective molecule [15]. Its lack of erythropoietic activity was determined using an in vivo hematopoiesis rodent model [15]. ARA 284 was provided by ARAIM Pharmaceuticals, Tarrytown, NY, USA.

### 2.3. Treatment with Bisoprolol and ARA 284

The dosages employed were based on earlier in vivo studies that have shown tissue-protective effects of bisoprolol [11] and a non-hematopoietic, tissue-protective EPO derivative, ARA 284 [15], in a rat model of cancer cachexia. We used 25% or 75% of the most effective single dose of each compound. Rats were randomly allocated to control (non-tumor-bearing; *n* = 15) or to one of five treatment arms for tumor-bearing animals: (1) 5 mg/kg/day bisoprolol (*n* = 23), (2) 1.7 µg/kg/day ARA 284 (*n* = 22), (3) 25% (1.25 mg/kg/day bisoprolol + 0.425 µg/kg/day ARA 284) combination (*n* = 12), (4) 75% (3.75 mg/kg/day bisoprolol + 1.275 µg/kg/day ARA 284) combination (*n* = 9), or (5) placebo (*n* = 78). Treatment was started on Day 1 after tumor inoculation. Six rats were excluded from the analysis, because of an ascites volume below 75 mL (ARA 284: *n* = 1, high dose combination: *n* = 3, placebo: *n* = 2).

### 2.4. Body Composition

The body composition of rats was examined as previously discussed using NMR scans (EchoMRI-700, Echo Medical Systems, Houston, TX, USA) [16].

### 2.5. Food Intake and Spontaneous Activity

Individually housed rats were given 100 g of food and 300 mL of water, and their movement and food intake were continuously monitored in all rats for a 24-h period before tumor inoculation and again on Day 10/11 before echocardiography using an infrared scanner with the Supermax activity monitoring system (Muromachi Kikai Co., Ltd., Tokyo, Japan) [17].

### 2.6. Cardiac Function

Echocardiography was performed at both the baseline and Day 11, as previously mentioned [18]. Echocardiographic parameters were measured, including LVEF (left ventricular ejection fraction), LVFS (left ventricular fractional shortening), LVEDV (left ventricular end-diastolic volume), LVESV (left ventricular end-systolic volume), LVSV (left ventricular stroke volume), LVmass (left ventricular mass), LVEDD (left ventricular end-diastolic diameter), and LVESD (left ventricular end-systolic diameter). Rats were briefly anaesthetized using 1.5% isoflurane. The body temperature was measured and maintained between 36 and 38 °C using a heating pad. All hair was removed from the chest using a chemical hair remover. The midline of the chest was drawn and divided into three equal segments, and the inlet and inferior limits of the thorax were indicated. These studies used a high-resolution echocardiography system (Vevo 770; VisualSonics Inc., Toronto, ON, Canada).

### 2.7. Statistical Analysis

Data analysis was performed using GraphPad PRISM 8.0 (GraphPad Software, Inc., La Jolla, CA, USA). The results were presented as mean ± SEM. Normal distribution was analyzed for all data using the Kolmogorov–Smirnov test. Within the control groups or tumor-bearing groups, data were analyzed according to their distribution. First, control animal data were compared to tumor-bearing animals using a *t*-test. Within each group, normally distributed data were analyzed using analysis of variance and Dunnett’s multiple comparisons test, whereas non-normally distributed data were analyzed using Kruskal-Wallis and Dunn’s multiple comparisons test. Survival was tested by Cox-proportional hazard analysis, which provides hazard ratios with 95% confidence intervals. Statistical significance was defined as a *p* value < 0.05 (two-tailed). No formal a priori sample size calculation was performed. The group sizes were determined based on previous experience with the Yoshida hepatoma cachexia model, ethical considerations to reduce the total number of animals, the need to include placebo-treated tumor-bearing animals across different tumor inoculation days, and the limited availability of ARA 284. Therefore, the present study should be regarded as an exploratory proof-of-concept study. Group sizes differed between endpoints because technically valid individual measurements were not available for all animals in all analyses. Therefore, the number of animals included varied depending on the availability of valid data for each specific endpoint.

## 3. Results

Tumor size, measured as ascites volume on the day of euthanasia, did not differ significantly between the groups and was 123 ± 25 mL in the placebo group, 112 ± 38 mL in the 5 mg bisoprolol group, 107 ± 33 mL in the high-dose ARA 284 group, 112 ± 38 mL in the 25% combination group, and 108 ± 58 mL in the 75% combination group. Tumor cell numbers were 2.99 ± 0.27 × 10^9^ for placebo, 3.91 ± 0.38 × 10^9^ for 5 mg bisoprolol, 2.62 ± 0.42 × 10^9^ for ARA 284, 2.53 ± 0.54 × 10^9^ for the 25% combination, and 3.35 ± 0.55 × 10^9^ for the 75% combinations groups (all *p* > 0.35).

### 3.1. Survival During the Study Period

**Figure 1:**
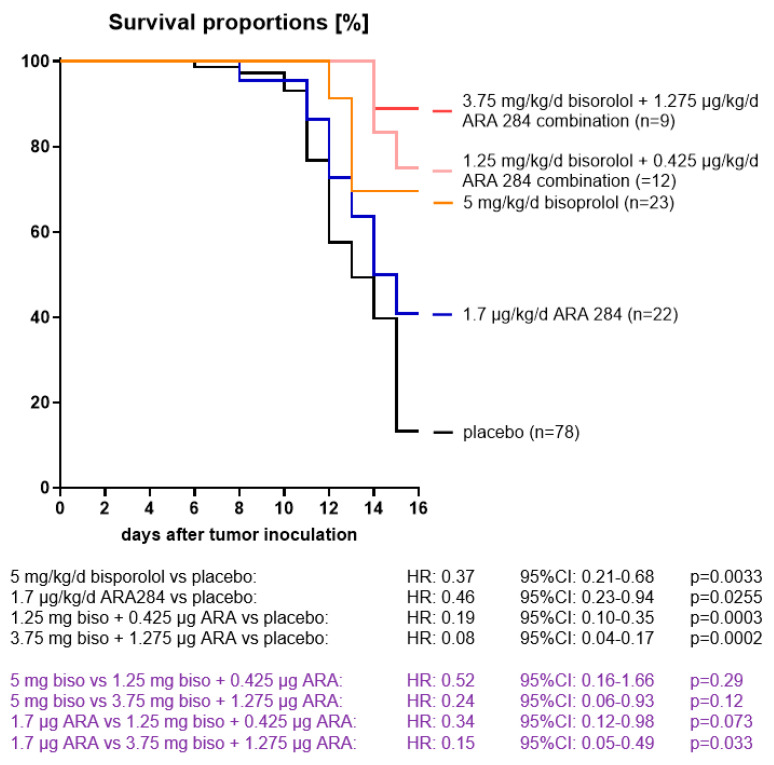
Combination therapies improved survival compared to the individual compounds.

The combination of 3.75 mg/kg/day bisoprolol and 1.275 µg/kg/day ARA 284 had the strongest effects on survival against the placebo (hazard ratio [HR] 0.08, 95% confidence interval [CI] 0.04 to 0.17, *p* = 0.0002), although 1.25 mg bisoprolol and 0.425 µg ARA combination and 5 mg bisoprolol alone had also a significant effect against the placebo (HR 0.19, 95% CI 0.10 to 0.35, *p* = 0.0003; and HR 0.37, 95% CI 0.21 to 0.68, *p* = 0.0033, respectively) (Figure 1).

### 3.2. Body Weight and Body Composition

**Figure 2:**
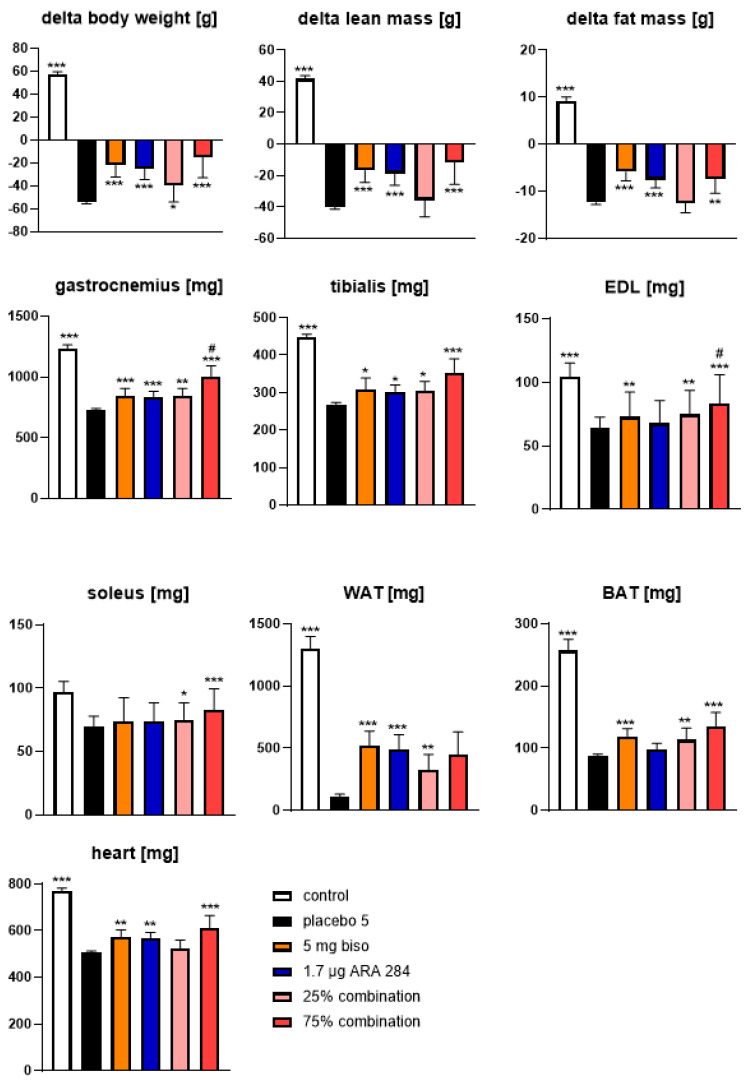
All intervention groups attenuated weight loss. The high-dose combination showed the most consistent overall effects on lean mass and tissue weight preservation, although the strongest effects on fat mass were observed in the bisoprolol and ARA 284 monotherapy groups. EDL: extensor digitorum longus, WAT: white adipose tissue, BAT: brown adipose tissue. *: *p* < 0.05, **: *p* < 0.01, ***: *p* < 0.001 vs. placebo, #: *p* < 0.05 vs. 1.7 µg/kg/day ARA 284. Organ weight was assessed at the end of the study or on the day of euthanasia, after reaching ethical endpoints. All animals were included in the analysis, irrespective of the day of euthanasia.

There were no significant differences for body weight, fat, and lean body mass at baseline, while significant improvements in body weight and lean body mass were observed in the 5mg/kg/day bisoprolol, the 1.7 µg/kg/day ARA 284, and the 75% combination at the end of the study. Fat mass was only improved in the 75% combination group and the 25% combination showed no improvement compared with placebo (Supplementary Materials, Table S1). Daily treatment with the combination of 3.75 mg/kg/day bisoprolol and 1.275 µg/kg/day ARA 284 had the most efficient effects for attenuation of body weight and lean mass compared with the placebo (*p* < 0.001) (Figure 2). In contrast, on attenuation of fat mass, treatment with 5 mg/kg/day bisoprolol and 1.7 µg/kg/day ARA 284 showed highly significant effects compared with the placebo (*p* < 0.001 vs. placebo, respectively).

The weights of the gastrocnemius, soleus, tibialis, and extensor digitorum longus (EDL) muscle were maximal in animals treated with the combination of 3.75 mg/kg/day bisoprolol and 1.275 µg/kg/day ARA 284 compared with the placebo (*p* < 0.001, respectively) (Figure 2). In the weight of the gastrocnemius and EDL, this combination treatment was also beneficial against 1.7 µg/kg/day ARA 284 alone. Notably, the 5 mg/kg/day bisoprolol maintained white adipose tissue (WAT) and the combination of 3.75 mg/kg/day bisoprolol and 1.275 µg/kg/day ARA 284 maintained brown adipose tissue (BAT).

### 3.3. Food Intake and Spontaneous Activity

**Figure 3:**
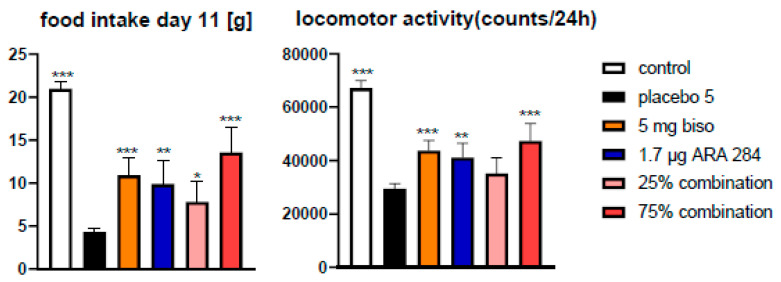
Effects of the bisoprolol and ARA 284 combination on food intake and spontaneous activity. *: *p* < 0.05, **: *p* < 0.01, ***: *p* < 0.001 vs. placebo. Group sizes were: 15 for sham, 68 for placebo, 23 for 5 mg/kg/day bisoprolol, 21 for 1.7 µg/kg/day ARA 284, 12 for low dose combination, and 9 for high dose combination at the time of the analysis.

The combination of 3.75 mg/kg/day bisoprolol and 1.275 µg/kg/day ARA 284 resulted in the greatest increase in food intake and locomotor activity compared with placebo (*p* < 0.001) (Figure 3).

### 3.4. Cardiac Function

The combination of 3.75 mg/kg/day bisoprolol and 1.275 µg/kg/day ARA 284 resulted in significant preservation of cardiac mass, leading to higher heart weight (Figure 2) compared with the placebo on the day of euthanasia (*p* < 0.001) (Table 1). In addition, this combination treatment had significant effects on left ventricular (LV) mass, LV ejection fraction, LV fractional shortening, LV end-diastolic volume, and LV stroke volume on Day 11 compared with placebo, whereas neither combination treatment had a significant effect on LV end-systolic volume. The combination of 1.25 mg/kg/day bisoprolol and 0.425 µg/kg/day ARA 284 had no significant effect on heart weight and cardiac functions compared with the placebo.

## 4. Discussion

We found that the combination of 3.75 mg/kg/day bisoprolol and 1.275 µg/kg/day ARA 284 showed the most consistent benefits across several major outcomes, including survival, lean mass, spontaneous activity, and cardiac function, in a rat model of cancer cachexia. These results suggest that combined targeting of β-blockade-related and tis-sue-protective pathways may provide enhanced therapeutic benefit in attenuating the multifactorial wasting process characteristic of cancer cachexia.

Bisoprolol has been reported to exert beneficial effects on body weight, body composition, cardiac function, and mortality in cancer cachexia, potentially through mechanisms involving modulation of Akt-related signaling and attenuation of sympathetic overactivation [11]. In the present study, these pathways were not directly assessed; therefore, these mechanistic interpretations remain speculative and are based on previous literature. Nevertheless, our physiological findings are consistent with the hypothesis that β-adrenergic modulation may contribute to preservation of muscle mass and cardiac function in cancer cachexia. Prior research has found that among the proteins studied, Akt is key in control-ling protein synthesis [11,19]. The previous research revealed that elevated Akt activity has a correlation with an increase in protein synthesis and simultaneous deactivation of proteins responsible for protein breakdown, as well as suppression of myogenesis [19,20]. Although such mechanisms were not directly analyzed in this study, our findings are consistent with the hypothesis that modulation of β-adrenergic signaling and Akt activation together may preserve muscle mass and cardiac integrity. In addition, patients and mice with cachexia have higher sympathetic tone, and prolonged increases in basal metabolic rate have been identified as a key mechanism for energy consumption in cancer cachexia [21,22]. Individuals suffering from heart failure show a noticeable activation of the sympathetic nervous system, and the plasma levels of norepinephrine are even more elevated in heart failure patients with cachexia compared to those without muscle loss [23]. Elevated activity of the sympathetic nervous system may play a role in cachexia by boosting overall energy expenditure in the body and by having a direct toxic impact on skeletal muscle tissue [24,25,26]. β-blockers can attenuate these conditions associated with sympathetic hyperactivity [27,28]. Thus, bisoprolol may act not only as a cardio-protective agent but also as an anti-cachectic intervention by counteracting sympathetic-driven metabolic overactivation in the cancer cachexia setting. In previous clinical trial, carvedilol, β-adrenergic blocking drug, demonstrated that not only significantly decreased the likelihood of further weight loss, but also resulted in a significant improvement of cachexia in a considerable number of patients with advanced left ventricular dysfunction [12]. In addition, espindolol, a novel non-selective β-blocker with central 5-HT1A and partial β2 receptor agonist effects, reversed weight loss, increased lean body mass, and improved handgrip strength compared with placebo in patients with advanced colorectal cancer and non-small cell lung cancer-related cachexia [29]. More recent preclinical studies have also supported the potential relevance of β-adrenergic modulation in cancer cachexia. For example, the atypical β-blocker S-oxprenolol reduced cachexia and improved survival in experimental cancer cachexia models [30], while ACM-001 improved cardiac function in a rat model of cancer cachexia-induced cardiomyopathy [31]. These findings, together with our results, support the rationale for β-blocker-based therapeutic strategies to mitigate cancer-induced wasting and cachexia-associated cardiac dysfunction.

ARA 284 has been reported to exert tissue-protective effects through pathways in-volving Akt activation, inhibition of p38 MAPK, and downregulation of myostatin in previous studies. However, these molecular pathways were not directly examined in the pre-sent study. Therefore, the current findings should not be interpreted as direct mechanistic evidence, but rather as being consistent with previously proposed tissue-protective mechanisms of ARA 284. High-dose ARA 284 increased the phosphorylation of Akt at the position Ser473 [15], thereby activating it inducing protein synthesis downstream [32]. In addition, ARA 284 decreased the levels of p38 MAPK phosphorylation and myostatin. The activation of p38 MAPK by cytokines such as TNF-α has been demonstrated to result in the activation of protein degradation through the up-regulation of the E3 ubiquitin ligases MAFbx and MuRF-1 [33]. The level of the phosphorylated form of p38 MAPK (Thr180/Tyr182) has been shown to rise during muscle wasting conditions linked to a range of diseases, including acute quadriplegic myopathy and type II diabetes, as well as ageing [34,35]. Myostatin, known for its role as a negative regulator of muscle growth, can activate several signaling pathways in skeletal muscle cells, including activation of p38 MAPK, which can result in muscle wasting [36,37]. While these molecular mechanisms have been described in previous reports, our current findings extend this knowledge by demonstrating that ARA 284, in combination with bisoprolol, translated these protective effects into improved whole-body outcomes (lean and fat mass preservation, cardiac function, and survival) in cancer cachexia.

Bisoprolol and ARA 284, when administered together at the effective combination dose, showed efficacy in the Yoshida hepatoma rat model of cancer cachexia. Our previous studies have already demonstrated dose-dependent effects of these two drugs separately on body weight, body composition, cardiac function, and mortality [11,15]. Among the five treatments including the placebo administered in this study, the treatment group containing 75% each of bisoprolol and ARA 284 may have shown the most pronounced effective effects against cancer cachexia. This dose combination likely reached the pharmacological threshold required to induce meaningful anti-cachectic effects, whereas the lower-dose combination remained subtherapeutic. The rationale for combining bisoprolol and ARA 284 is that cancer cachexia is a multifactorial syndrome involving skeletal and cardiac muscle wasting, anorexia, reduced activity, and metabolic dysregulation. Therefore, simultaneous targeting of β-adrenergic and tissue-protective pathways may offer broader benefit than either approach alone. Importantly, the observed benefits are unlikely to be explained solely by delayed tumor progression, as ascites volume and tumor cell number did not differ significantly between groups. This suggests that the improvements in body weight, lean mass, activity, cardiac function, and survival may reflect modification of cachexia-related processes rather than a simple reduction in tumor burden. However, the present study does not fully separate direct anti-cachectic effects from a broader influence on disease progression, and this distinction should be addressed more directly in future studies. Cancer cachexia, a multifactorial syndrome that includes a progressive loss of skeletal muscle, wasting of adipose tissue, anorexia, asthenia, systemic inflammation, and functional deterioration, is linked to higher morbidity and death in cancer patients [38,39]. A previous study reported that low muscle index, low muscular attenuation, and high weight loss all independently predicted survival in patients with cancer cachexia [40,41,42]. In addition, cachexia affects multiple organ systems, including the heart, and cardiac wasting and heart failure may substantially contribute to morbidity and mortality in patients with cancer [43,44,45]. Recent clinical evidence has further demonstrated that cardiac wasting is clinically relevant in patients with advanced cancer and is associated with impaired functional status and poor prognosis [46]. Patients with cachexia often suffer from cardiac atrophy and dysfunction, which may contribute to the pathogenesis and progression of cachexia [44]. Cardiac wasting may also contribute to ventricular arrhythmias and heart failure, thereby worsening the prognosis of patients with cancer cachexia [45]. Our results confirm these associations experimentally and indicate that the combination therapy effectively counteracted both skeletal and cardiac muscle wasting in this cancer cachexia model. Because body composition was assessed by NMR, which distinguishes lean mass from fat mass, the preservation of skeletal muscle weights observed in the treatment groups is unlikely to be explained solely by reduced fat loss. However, muscle weight alone does not provide direct histological evidence of anti-atrophic effects. As muscle fiber cross-sectional area was not assessed in the present study, our findings should be interpreted as indicating preservation of lean and muscle mass rather than definitive proof of myofiber-level anti-atrophic effects. Importantly, the discussion should be placed within the context of cancer cachexia rather than cardiac cachexia, as our rat model primarily reflects cancer-associated metabolic and structural deterioration. Therefore, the combination therapy of bisoprolol and ARA 284 may have a positive impact on body composition and cardiac function, and improve prognosis in clinical trials. These findings emphasize the translational relevance of targeting both metabolic (via ARA 284) and neurohumoral (via bisoprolol) pathways in the management of cancer cachexia. In contrast, the low-dose combination (1.25 mg/kg bisoprolol + 0.425 µg/kg ARA 284) was likely subtherapeutic, failing to reach the threshold needed to elicit significant physiological benefits. Correspondingly, this group showed no measurable improvement in cardiac parameters (heart weight or systolic function) nor any preservation of lean/fat mass or spontaneous locomotor activity. Such a lack of effect suggests a non-linear dose–response (threshold) phenomenon, wherein only sufficiently high drug exposures produce meaningful anti-cachectic outcomes. This interpretation is supported by prior studies showing that bisoprolol’s cardioprotective efficacy in cachexia emerges only at higher doses (5 mg/kg) [11], and that ARA 284 likewise improves survival only at a higher dose [15]. In future studies, ARA 284 should possibly be administered several times per day or even as a continuous infusion using osmotic pumps. Accordingly, the low-dose regimen in our study likely fell below the efficacious dose range, whereas the high-dose combination surpassed this threshold to achieve robust benefits across measured end-points. However, the present study was not designed to perform a comprehensive dose-combination analysis. Instead, only two reduced-dose combination regimens based on previously established effective monotherapy doses were examined as an initial proof-of-concept approach. Therefore, the optimal balance between bisoprolol and ARA 284 cannot be determined from the current data alone. Future studies should investigate a broader range of dose combinations, including regimens with relatively higher ARA 284 and lower bisoprolol or vice versa, in order to better characterize dose–response relation-ships and identify the most effective combination strategy.

This study has several limitations. First, the sample sizes among groups were unbalanced, and no formal a priori sample size calculation was performed. The present study was designed as an exploratory proof-of-concept preclinical study based on previous experience with the Yoshida hepatoma cachexia model and previously established effective monotherapy doses of bisoprolol and ARA 284. The larger placebo group was used as a common reference group across different tumor inoculation days, because not all animals could be assessed in parallel and placebo-treated tumor-bearing animals were required on each experimental day to control for potential variability in tumor aggressiveness. In contrast, the smaller sample sizes in the ARA 284 and combination groups were partly due to limited compound availability and ethical considerations regarding the total number of animals used. This imbalance may reduce statistical precision, particularly in the smaller treatment groups, and may increase the uncertainty of effect estimates. Therefore, the pre-sent findings should be interpreted as exploratory and hypothesis-generating rather than confirmatory. Future studies with balanced group allocation and prospective sample size calculation are required to confirm these results. Second, histological assessment of skeletal muscle, including muscle fiber cross-sectional area, was not performed. Therefore, although the treatment groups showed preservation of lean body mass and muscle weights, direct confirmation of anti-atrophic effects at the muscle fiber level was not possible. Third, although this study evaluated anorexia, one of the systemic features of cachexia, by assessing food intake, anemia and inflammation were not examined. Further evaluation of these parameters may have enhanced the clinical relevance of our findings. Fourth, the experimental period of this study was relatively short (16 days), which limits the evaluation of long-term efficacy. Accordingly, the survival analysis was restricted to the predefined study period and should therefore be interpreted as survival during the observation period rather than long-term survival. Future studies with longer follow-up might provide a more comprehensive assessment of treatment effects on prognosis. Since cachexia in patients develops progressively over several months, future studies should examine the medium- to long-term effects using clinical investigations. For clinical application in patients with cachexia, pharmacological and safety concerns must be carefully addressed. While bisoprolol is already established in clinical practice, its use in patients with advanced cancer cachexia may require careful monitoring for bradycardia, hypotension, and reduced physiological reserve. In addition, the safety profile, pharmacological interactions, tolerability, and appropriate dosing strategy of ARA 284 in combination with bisoprolol remain to be established. Further studies are therefore warranted to investigate the safety, feasibility, and optimal dosing strategy of this combination before clinical translation. Additionally, mechanistic studies assessing molecular markers of anabolic and catabolic balance (e.g., Akt, p38 MAPK, myostatin) in future experiments could strengthen the link between physiological outcomes and the underlying signaling pathway. This study only used male rats, while a balanced number of male and female rats might have been advisable, but would have increased the total number of animals used in this study.

## 5. Conclusions

In conclusion, we demonstrated positive effects of the combination of bisoprolol and ARA 284 on body composition, cardiac function, and mortality in a rat model of cancer cachexia. These findings highlight a promising synergistic approach for treating cancer cachexia, integrating modulation of sympathetic activity and tissue-protective signaling. Future pre-clinical studies are warranted to further define the translational potential of this combined therapy.

## Figures and Tables

**Table 1:** Effects of bisoprolol and ARA 284 combination therapy on cardiac function and left ventricular structure.

	Control	Placebo	5 mg/kg/day Bisoprolol	1.7 µg/kg/day ARA 284	25% Combination	75% Combination
Baseline LVEF %	72.16 ± 2.38	76.84 ± 0.85	74.72 ± 1.79	70.74 ± 1.73	72.17 ± 2.05	75.03 ± 1.84
Day 11 LVEF %	72.8 ± 9.1 ***	54.3 ± 14.3	57.1 ± 15.4	57.2 ± 22	54.4 ± 13.6	63.6 ± 9.9 *
Delta LVEF %	3.0 ± 8.8 ***	−25.5 ± 14.9	−20.8 ± 15.1	−14.0 ± 14.0 *	−17.7 ± 14.4	−11.4 ± 9.3 *
Baseline LVFS %	48.00 ± 1.70	50.86 ± 0.86	48.00 ± 1.41	47.38 ± 1.40	47.21 ± 1.65	50.57 ± 2.58
Day 11 LVFS %	51.6 ± 5.9 ***	31.3 ± 10.0	34.7 ± 9.9	38.8 ± 16.7 *	35.0 ± 12.1	42.2 ± 9.6 *
Delta LVFS %	3.5 ± 5.9 ***	−19.6 ± 12.4	−16.5 ± 10.9	−9.2 ± 15.9 *	−12.2 ± 14.3	−8.4 ± 8.5 *
Baseline LVEDV µL	242.57 ± 13.34	254.45 ± 4.48	266.18 ± 9.62	229.83 ± 7.82	275.58 ± 7.87	272.97 ± 9.64
Day 11 LVEDV µL	269.2 ± 41.2 **	189.6 ± 59.1	262.0 ± 114.4 **	238.6 ± 81.8 *	227.6 ± 68.3	285.9 ± 68.7 **
Delta LVEDV µL	32.1 ± 60.2 **	−68.8 ± 66.9	−10.3 ± 114.3 *	5.3 ± 104.4 **	−48.9 ± 68.7	25.4 ± 88.4 **
Baseline LVESV µL	65.91 ± 5.37	59.83 ± 2.39	66.87 ± 5.04	69.65 ± 3.52	66.39 ± 5.27	77.54 ± 7.38
Day 11 LVESV µL	70.8 ± 22.9 **	99.1 ± 22.1	86.4 ± 26.3	89.4 ± 28.4	98.1 ± 24.1	93.5 ± 15.2
Delta LVESV µL	2.8 ± 22.4 **	40.1 ± 25.4	35.2 ± 32.7	23.6 ± 33.6	20.6 ± 39.6	25.5 ± 23.7
Baseline LVSV µL	176.66 ± 12.82	196.54 ± 3.95	199.32 ± 8.94	166.22 ± 7.65	198.05 ± 6.27	201.63 ± 10.85
Day 11 LVSV µL	196.1 ± 39.0 ***	110.8 ± 53.5	170.8 ± 73.2 **	142.3 ± 89.9	128.6 ± 67.2	185 ± 62.6 **
Delta LVSV µL	29.4 ± 54.1 ***	−95.3 ± 60.3	−39.6 ± 78.7 *	−25.9 ± 96.9 **	−69.5 ± 54.8	−0.7 ± 68.8 **
Baseline LVmass mg	536.8 ± 116.5	527.4 ± 105.3	474.8 ± 69.6	534.9 ± 56.7	523.7 ± 36.4	527 ± 31.2
Day 11 LVmass mg	625.6 ± 138.4 ***	412.8 ± 54.3	469.8 ± 67.6	475.4 ± 105.4 *	439.0 ± 96.5	496.2 ± 65.5 *
Delta LVmass mg	110.1 ± 96.4 ***	−127.0 ± 68.7	−4.5 ± 112.6 **	−58.4 ± 119.9	−79.1 ± 114.0	−23.7 ± 62.1 *
Baseline LVEDD mm	6.22 ± 0.13	6.25 ± 0.05	6.45 ± 0.10	6.35 ± 0.09	6.47 ± 0.06	6.48 ± 0.09
Day 11 LVEDD mm	6.39 ± 0.31 **	5.67 ± 0.67	6.33 ± 1.12 *	6.03 ± 0.45	5.97 ± 0.57	6.38 ± 0.63 *
Delta LVEDD mm	0.26 ± 0.45 **	−0.66 ± 0.81	−0.06 ± 0.88 *	−0.24 ± 0.67	−0.47 ± 0.68	0.17 ± 0.45 *
Baseline LVESD mm	3.23 ± 0.13	3.08 ± 0.06	3.30 ± 0.12	3.34 ± 0.10	3.20 ± 0.17	3.42 ± 0.13
Day 11 LVESD mm	3.10 ± 0.48 ***	4.14 ± 0.47	3.96 ± 0.42	3.65 ± 0.95 *	3.87 ± 0.78	3.54 ± 0.31 *
Delta LVESD mm	−0.09 ± 0.53 ***	1.02 ± 0.62	0.81 ± 0.46	0.26 ± 0.89 **	0.45 ± 0.99 *	0.35 ± 0.46 *

Data are presented as mean ± SEM. LVEF: left ventricular ejection fraction, LVFS: left ventricular fractional shortening, LVEDV: left ventricular end-diastolic volume, LVESV left ventricular end-systolic volume, LVSV: left ventricular stroke volume, LVmass: left ventricular mass, LVEDD: left ventricular end-diastolic diameter, LVESD: left ventricular end-systolic diameter. *: *p* < 0.05, **: *p* < 0.01, ***: *p* < 0.001 vs. placebo. Group sizes were: 15 for sham, 56 for placebo, 23 for 5 mg/kg/day bisoprolol, 19 for 1.7 µg/kg/day ARA 284, 12 for low dose combination, and 9 for high dose combination at the time of the analysis.

## Data Availability

The data presented in this study are available on request from the corresponding author.

## Supplementary Materials

The following supporting information can be downloaded at: https://www.mdpi.com/article/10.3390/jcdd13060241/s1, Table S1: Baseline and final body weight and body composition (fat and lean mass).
