# Supplementary material for: Combination Therapy with Bisoprolol and Tissue Protective Molecule ARA 284 Is Cardio-Protective and Improves Survival in Experimental Cancer Cachexia
Source: J Cardiovasc Dev Dis. 2026 Jun 1;13(6):241. doi: 10.3390/jcdd13060241 (PMC13301489; doi:10.3390/jcdd13060241)
Supplement: Supplementary file 1 [file jcdd-13-00241-s001.zip › jcdd-4225608-supplementary.pdf]

Supplementary Materials

Table S1. Baseline and final body weight and body composition (fat and lean mass)

	Control	Placebo	5 mg/kg/day bisoprolol	1.7 µg/kg/day ARA 284	25% combination	75% combination
Body weight BL	202 ± 4	206 ± 1	204 ± 2	204 ± 2	213 ± 2	214 ± 2
Body weight end	263 ± 4***	151 ± 2	180 ± 11***	187 ± 12***	172 ± 14	199 ± 17***
Lean mass BL	157.9 ± 2.9	158.3 ± 1.0	157.6 ± 1.8	156.2 ± 1.3	163.9 ± 2.0	165.6 ± 2.0
Lean mass end	199.9 ± 3.7***	118.5 ± 1.6	141.0 ± 8.3**	137.5 ± 7.8*	128.5 ± 10.9	156.0 ± 13.4***
Fat mass BL	15.6 ± 0.6	18.7 ± 0.4	16.7 ± 0.6	20.4 ± 0.8	20.8 ± 0.5	20.5 ± 1.2
Fat mass end	24.3 ± 0.8***	6.4 ± 0.4	11.1 ± 1.8	12.9 ± 2.1	8.7 ± 2.1	13.1 ± 3.1*

All data are presented in grams. BL: baseline; end: end of study. *: $p < 0.05$, **: $p < 0.01$, ***: $p<0.001$ vs. placebo. Body weight and body composition data were analyzed after tumor removal, irrespective of the day of euthanasia.
